# Comparing the Performances of Chimpanzees (*Pan troglodytes*) and Gorillas (*Gorilla gorilla gorilla*) in Two Self‐Awareness Tasks

**DOI:** 10.1002/ajp.70010

**Published:** 2025-03-06

**Authors:** Lisa‐Claire Vanhooland, Constanze Mager, Aurora Teuben, Thomas Bugnyar, Jorg J. M. Massen

**Affiliations:** ^1^ Department of Behavioral and Cognitive Biology University of Vienna Vienna Austria; ^2^ Animal Behaviour and Cognition Utrecht University Utrecht the Netherlands; ^3^ Royal Burgers´ Zoo Arnhem the Netherlands; ^4^ University of Amsterdam Amsterdam the Netherlands

**Keywords:** body image, body schema, body self‐awareness, body‐as‐obstacle task, mirror self‐recognition

## Abstract

Self‐awareness has most commonly been studied in nonhuman animals by implementing mirror self‐recognition (MSR) tasks. The validity of such tasks as a stand‐alone method has, however, been debated due to their high interindividual variation (including in species deemed self‐aware like chimpanzees), their reliance on only one sensory modality, their discrete outcomes (i.e., pass/fail) and, in general, questionned regarding their ability to assess self‐awareness. Therefore, a greater variety of methods that assess different aspects of the self, while simultaneously contributing to a more gradualist view of self‐awareness, would be desirable. One such method is the body‐as‐obstacle task (BAO), testing for another dimension of body self‐awareness. The ability to understand one's own body as an obstacle to the completion of a desired action emerges in young children at approximately the same age as mirror self‐recognition, suggesting a shared mental representation. Whereas recently some studies showed body self‐awareness in nonhuman animals, so far, outside of children no studies have compared how the performances of individuals relate between these two tasks. Therefore, here we study both a MSR and a BAO task in chimpanzees and gorillas. We chose these species particularly because evidence for MSR in chimpanzees is well established, whereas results for gorillas have been mixed, which has been attributed to the study design of MSR tasks, and for which a BAO task might thus provide more conclusive evidence. We find that although only some chimpanzees showed evidence for mirror self‐recognition, thus replicating previous findings on interspecies differences in MSR, chimpanzees and gorillas performed equally well in the BAO task. Yet, we further found no correlation between the individuals' performances in both tasks. We discuss the implications of these findings for the interpretation of the results of BAO tasks as a possible alternative paradigm for the study of self‐awareness in non‐human animals.

## Summary

- Gorillas and chimpanzees perform equally well in a body‐as‐obstacle task, despite only a few chimpanzees exhibiting signs of mirror self‐recognition
- We find no correlation between the individuals' success rates in the two self‐awareness task

## Graphical abstract

Here we study the performances of chimpanzees and gorillas in two body self‐awareness tasks: a mirror self‐recognition task and a body‐as‐obstacle task. We find that although only some chimpanzees showed evidence for mirror self‐recognition, chimpanzees and gorillas performed equally well in the BAO task. We found no correlation between the individuals' performances in both tasks. We discuss the implications of these findings for the interpretation of the results of BAO tasks as a possible alternative paradigm for the study of self‐awareness in non‐animals.

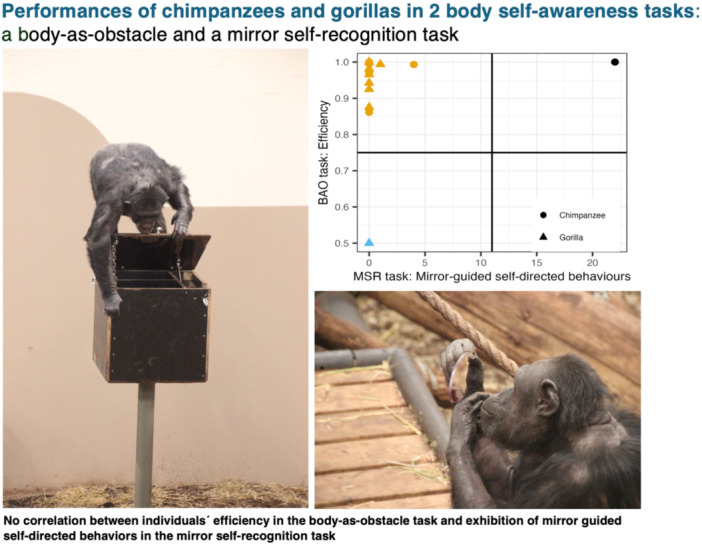

## 1 Introduction

Self‐awareness (i.e., the concept and sense of self and the ability to become the object of one's own attention) is a multifaceted construct that allows individuals to become aware of their own identity, thoughts, feelings, and behaviors (Morin 2006). Self‐awareness is theorized to be a stepping stone towards other advanced cognitive abilities, such as Theory of Mind and empathy, by attributing the same self‐knowledge abilities to others (Gallup 1982; Gallup 1998) and to facilitate the navigation of the social environment, e.g., through self‐regulation (Morin 2011). Studying self‐awareness in nonhuman animals, therefore, holds the potential to further our understanding of mental states in animals and the mechanisms underlying the development of self‐awareness. In humans, the ability to recognize oneself in a mirror generally emerges between 18 and 24 months of age in toddlers raised in Western cultures (Amsterdam 1972; Lewis and Brooks‐Gunn 1979, but see: Broesch et al. 2011; Cebioğlu and Broesch 2021; Kärtner et al. 2011, 2011; Keller et al. 2004 for discussions on cultural variations in MSR) and is accompanied by the development of pretend play, mimicry, the use of personal pronouns, self‐conscious emotions, empathic concern, and secondary representations (Bischof‐Köhler 2012; Nielsen and Dissanayake 2004), all indicative of a crystallizing concept of self and objective self‐awareness in the second year of life.

To study the evolution of self‐awareness, a phylogenetic approach, exploring and comparing the self‐awareness abilities in other species, is necessary and requires adequate testing methods. Yet, most assessments of aspects of self‐awareness in humans rely on self‐reports (Morin 2011) that inquire about the subjective experience of an individual, a dimension generally not accessible when working with nonhuman animals.

In nonhuman animals, self‐awareness has most commonly been studied by exploring an individual's visual self‐recognition abilities in the form of mirror self‐recognition. Mirror self‐recognition explorations are often coupled with the implementation of a mark test, in which marks are inconspicuously applied to an individual on a body part that it would not be able to explore without the use of a mirror. Attempts to explore normally non‐visible body parts in the mirror and subsequent attempts to remove these markings are considered clear indications of mirror self‐recognition (Gallup 1970).

As humans' closest living relatives, nonhuman primates have been at the center of investigations into MSR, particularly after Gordon Gallup's seminal study in 1970, in which he showed that chimpanzees, in contrast to three macaque species, show signs of MSR and successfully pass the mark test. This dichotomy between the performances of great apes and monkeys was further substantiated by later studies (Gallup 2002; Inoue‐Nakamura 1997). Although monkeys appear capable of using a mirror to gain visual access to places, objects, or conspecifics that they are not able to see due to obstructions (Anderson 1986; Anderson et al. 2021; Itakura 1987), they fail to show signs of self‐recognition when exposed to their reflection. Contrarily, the ability of great apes to recognize themselves in mirrors, with the exception of gorillas, has by now been well established (Gallup 1970; Gallup et al. 1995; Lethmate and Dücker 1973; Murray et al. 2022; Suarez and Gallup 1981).

There are two main, and non‐mutually exclusive, hypotheses about why self‐awareness might have emerged in great apes and not in monkeys. The first is the arboreal clambering hypothesis, advanced by Povinelli (1995), which hypothesizes that the concept of “self” developed in the ancestor of great apes, due to a need stemming from navigating arboreal living with large and heavy bodies (Povinelli 1995). While the smaller and lighter monkeys could continue to rely on stereotyped body movements for moving through the trees, the larger‐bodied apes were required to develop specific knowledge and a representation of their own bodies while maneuvering through the canopy (Povinelli 1995). Others propose the emergence of self‐awareness and the subsequent awareness of others' mental states as the result of navigating a complex social environment, and a combined need for competition and cooperation with conspecifics (Byrne and Whiten 1988; Dunbar 1998; Humphrey 1976).

Despite an ongoing debate about what MSR experiments are actually measuring—i.e., whether they reflect the possession of a mental model of self, or whether they might be explained through simpler mechanisms (de Waal 2019; Heyes 1994, 1996; Kohda et al. 2019; Loveland 1986; Mitchell 1993, 1995; Suddendorf and Butler 2013, 2014)—the mirror self‐recognition task remains, to date, the predominant way of testing for self‐awareness in nonhuman animals. Due to their pass‐or‐fail outcome, the results of mirror self‐recognition tests are, however, insensitive to possible gradients of self‐awareness (de Waal 2019; Rochat 2003). As a result of this one‐test approach, self‐awareness continues to be conceived as unidimensional, leading to the neglect of other constituent components of self‐awareness and prohibiting the full exploration of self‐awareness, a multifaceted construct, which might not be composed of the same building blocks in all species (Lage et al. 2022). Therefore, a more diverse approach to the measurement of self‐awareness, by diversifying the types of implemented tests and finding alternatives to the MSR task for studying self‐awareness, would be desirable, particularly when studying self‐awareness in nonhuman animals.

One such approach is the assessment of an individual's ability to represent their body's physical characteristics, such as weight or size (Brownell et al. 2007), which explores other dimensions of body self‐awareness (BSA) than MSR tests. Contrarily to MSR tasks, such BSA tests have a higher ecological relevance, as knowledge about one's own body weight or size can be essential for survival during locomotion (i.e., not getting stuck in holes, not stepping on branches that wouldn't hold one's weight (cf. arboreal clambering hypothesis (Povinelli 1995)).

Studies in human infants show that the ability to represent those characteristics, i.e., one's own body size and one's own body as an obstacle, might be two distinct components of BSA (Brownell et al. 2007). Similarly to MSR, these abilities appear to be expressions of explicit self‐awareness that emerge in humans in the second half of the second year of life (Barth et al. 2004; Brownell et al. 2007; Moore et al. 2007). Body‐as‐obstacle tasks (BAO) typically aim to test the individual's understanding that their own body is the object standing in the way, preventing them from performing the task at hand. Examples of such tasks are the shopping cart task and the blanket task (Brownell et al. 2007; Moore et al. 2007).

In the shopping cart task, infants are asked to push a shopping cart, which has a blanket attached to its back, towards their parent. To reach for the cart's handle, the child necessarily steps on the blanket, thus blocking the movement of the cart through their own body weight (Brownell et al. 2007; Moore et al. 2007). Following the same logic, in the blanket task, an infant is placed on a blanket, which they are then asked to hand over (Brownell et al. 2007). In both cases, the child needs to understand the need to step off the blanket to successfully perform the requested and intended action.

In a study conducted on 18‐month‐old children confronted with a MSR and a BAO task, Moore and colleagues (2007) were able to show that both MSR and the ability to pass the BAO task seem to arise in parallel in infants. This poses the question of whether these two abilities are two different expressions of the same underlying mechanism, related to the integration and mental representation of one's own body image as suggested by the arboreal clambering hypothesis (Bullock and Lütkenhaus 1990; Moore et al. 2007). Yet, the relatively low correlation between both tasks might indicate that two distinct aspects of the objective self might be expressed in these tasks (Moore et al. 2007).

So far, the body size as well as BAO components of BSA have only been investigated in a handful of nonhuman species (elephants: Dale and Plotnik 2017; snakes: Khvatov et al. 2019; rats and ferrets: Khvatov et al. 2021b, 2021a; hooded crows: Khvatov et al. 2021; dogs: Lenkei et al. 2020, 2021; cats: Pongrácz 2024; Schiffner et al. 2014 ). In particular, a study in elephants (Dale and Plotnik 2017), a species that also passed the mirror mark test and thus shows MSR, has shown that similar to infants (Moore et al. 2007), they demonstrated an ability to understand their own body as a physical obstacle to the performance of a desired action. However, both human and animal studies relied on a close interaction between the tested individual and a human experimenter giving verbal commands e.g., the mother asking the child to push the cart towards her (Moore et al. 2007) or an elephant being asked to pick up a stick and bring it to the experimenter (Dale and Plotnik 2017), limiting the potential application of this test to trained animals with which such commands were previously established.

Although nonhuman primates, including the apes, have not been investigated in such BSA paradigms yet, the arboreal clambering hypothesis offers an interesting framework and testable hypothesis. This is especially relevant as the positive correlation between performances in the MSR and BAO task observed in children (Moore et al. 2007) has not yet been established in nonhuman animals.

Chimpanzees and gorillas are particularly interesting candidates for exploring the connection between different expressions of BSA considering the close phylogenetic relatedness of the two species (Satta et al. 2000), their similar cognitive abilities in self‐awareness related domains like Theory of Mind (Parker 1991) yet their different ontogenetic developments (Mitteroecker et al. 2004; Potì and Spinozzi 1994; Shea 1983; Watts and Pusey 1993), social structures (Maryanski 1987; Parnell 2002; M. J. Remis 1997; Symington 1990), propensities for arboreal lifestyles (M. Remis 1995; Reynolds 1965) and more importantly, capacities to pass MSR tasks (Inoue‐Nakamura 1997; Suarez and Gallup 1981). Chimpanzees have been particularly well‐studied regarding their self‐recognition abilities. Beyond their ability to recognize themselves in mirrors, chimpanzees have also shown the ability to recognize themselves in video recordings (Hirata et al. 2017), photographs (Anderson and Gallup 1999) and even seem to recognize their own shadows (Boysen et al. 1994), thus exhibiting evidence for several levels of explicit self‐awareness (Legrain et al. 2011; Rochat 2003). Moreover, although it has long been assumed that individuals must be exposed to a full‐body‐sized mirror (Plotnik et al. 2006) to learn the connection between themselves and their reflection, Kopp and colleagues (2021) recently showed that chimpanzees were not only capable of recognizing themselves in small handheld mirrors, but also engaged more with the smaller transportable mirrors in comparison to full‐body‐sized mirrors (Kopp et al. 2021).

The small mirrors elicited self‐directed behaviors from more individuals, and the chimpanzees spent more time exhibiting these behaviors with the small mirror than with a large mirror. Although the study (Kopp et al. 2021) did not include a control condition with non‐reflective handheld objects to see whether the chimpanzees' responses were mirror‐specific, it successfully addressed the common issue of high interindividual variations in performances and false negatives to which mirror self‐recognition tasks seem prone to, as on average, only a fourth to a third of tested nonhuman individuals successfully pass the test.

This novel methodology offers the additional benefits of preventing monopolization of the mirror and of prompting social behaviors—particularly aggressive reactions—towards their reflection. Strong social responses towards mirrors have been theorized to inhibit the process towards mirror self‐recognition (Mahovetz et al. 2016). Small mirrors, by preventing full‐sized reflections of individuals, may be perceived as less threatening than full‐sized reflections in the initial stages of mirror exploration, when the mirrored image is perceived as a conspecific. Consequently, smaller mirrors could be beneficial in the MSR process, especially in species known for very strong aggressive or avoidant social reactions towards mirrors. The lessened perceived threat arising from the “conspecific” in the mirror, which has been posed as an argument for why gorillas have difficulties passing the mirror mark test (see below), might allow for more exploration of the mirror and their own reflection, which are essential for acquiring self‐recognition.

Chimpanzees further show one of the highest likelihoods of all nonhuman species to pass MSR tasks and therefore appear ideal candidates to explore whether we can find a correspondence between MSR and the ability to pass a BAO task in nonhuman primates. In contrast to chimpanzees, gorillas appear to perform very poorly in mirror self‐recognition tasks (see Murray et al. 2022 for review), with evidence of self‐recognition found in only three individuals (Allen and Schwartz 2008; Patterson and Cohn 1981; Posada and Colell 2007).

Several hypotheses have been put forward to explain the poor performances in MSR tasks by gorillas (Gallup et al. 1980; Parker et al. 1994; Patterson and Cohn 1981; Povinelli 1987). The most common hypothesis addresses the tendency for gaze aversion in gorillas as a hindering factor for mirror self‐recognition. Yet, attempts by Shillito and colleagues (1999) to circumvent direct eye contact by using angled mirrors did not improve the gorillas' performances (Shillito et al. 1999). Povinelli (1994), on the other hand, suggests the secondary loss of MSR in gorillas to have been caused by a heterochronic shift (i.e., changes in the timings of development) of certain cognitive mechanisms during ontogeny. Alternatively, Gallup proposes that it might be a consequence of sexual selection due to which a presumably costly ability such as self‐awareness no longer provided a reproductive advantage after socioecological changes (leading to lower competition for resources and reproduction opportunities) in the gorillas (Gallup 1997).

Another approach would consist of considering the ability of self‐recognition of a polymorphic trait within and across species, in which case one could conceive this trait to have a different prevalence in different populations (Povinelli 1994). Accordingly, this trait might be highly prevalent in humans (*Homo sapiens*), moderately in chimpanzees (*Pan troglodyte*) but very rare in gorillas (*Gorilla gorilla*). Gallup (1997) proposes as further explanation for the observed prevalence of MSR ability in the population, that this trait (lost in gorillas) might be in the process of being lost in the chimpanzees (Gallup 1997).

Consequently, comparatively assessing the connection between different dimensions of BSA in these two great ape species appears particularly interesting. Therefore, the following study aimed to assess the correlation between the performances of chimpanzees and gorillas in a mirror self‐recognition and a BAO task. For this purpose, we first exposed both species to handheld mirrors and non‐reflective objects to assess the individuals' expression (or lack thereof) of mirror self‐recognition. Subsequently, we presented them with a BAO task based on the same principle as the BAO test used in infants (i.e., the individual's own body weight hinders the completion of a desired action) that did not require a verbal command. Based on the arboreal clambering hypothesis and the performances of infants in such tasks (Moore et al. 2007), we expected to find a positive correlation between the performances in the MSR and BAO tasks in the chimpanzees. We further expected the gorillas to perform poorly in the mirror self‐recognition task due to the potential secondary loss of this ability in this species. Even though gorillas are far less arboreal than chimpanzees, we hypothesized the ability to perceive their own bodies as obstacles to still be present in the species; and thus expected the gorillas to succeed in the BAO task.

## 2 Material and Methods

### 2.1 Study Population

Two social groups of apes with continuous full contact within groups participated in this study: a group of 15 adult chimpanzees (*Pan troglodytes*) (four males, eleven females; age range: 16–61 years) and a group of 11 (sub‐) adult western lowland gorillas (*Gorilla gorilla gorilla*) (five males, six females; age range: 8–32 years), mean age ± SD: Chimpanzees: 35.13 ± 10.78 years, Gorillas 15.18 ± 8.75 years (see Table S1 in Supporting for specifications). Both groups were housed at the Royal Burgers' Zoo (Arnhem, the Netherlands) and had extensively furnished indoor (265 m^2^ each) and naturalistic outdoor enclosures (chimpanzees: ca. 4000 m^2^; gorillas: ca. 3000 m^2^) with enrichment provided to them. During the experiments, the daily routine of the apes was maintained, including all the feedings (the apes' diet included monkey chow, seeds, fruits, and vegetables) and water was accessible ad libitum. Most individuals were naïve to the experimental setups presented in this study, except the older chimpanzees that had encountered full body‐sized mirrors provided to them as enrichment about 20 years before conducting the present study. Although all individuals participated in the MSR task, we had to exclude two individuals from some of the statistical analyses for the BAO task – one chimpanzee female (Jimmy) who unfortunately died of old age (61) between the data collection for the test box and the control box conditions (see below) of the BAO task and one gorilla female (N'Aika), that experienced health concerns in some of the conditions of control box session of the BAO task, which notably affected her participation in the task.

### 2.2 Ethics Declaration

The current experiments were conducted as part of the apes' (chimpanzees and gorillas alike) enrichment program at the Royal Burgers' Zoo. The Royal Burgers' Zoo is a member of the European Association of Zoos and Aquaria and, consequently, meets the legal and ethical regulations on captive animal welfare. The present study did not meet the definition of an animal experiment as mentioned in Article 1 of the Dutch “Experiments on Animals Act” due to its noninvasive nature and the absence of any potential discomfort for the animals. Participation in this study was voluntary. Therefore, this study was conducted in compliance with all relevant Dutch laws and in agreement with international and scientific standards and guidelines, leading the ethics committee of Utrecht University to waive the need for approval.

### 2.3 Data Collection

#### 2.3.1 Task 1: Mirror Self‐Recognition Task

##### 2.3.1.1 Material

**Figure 1:**
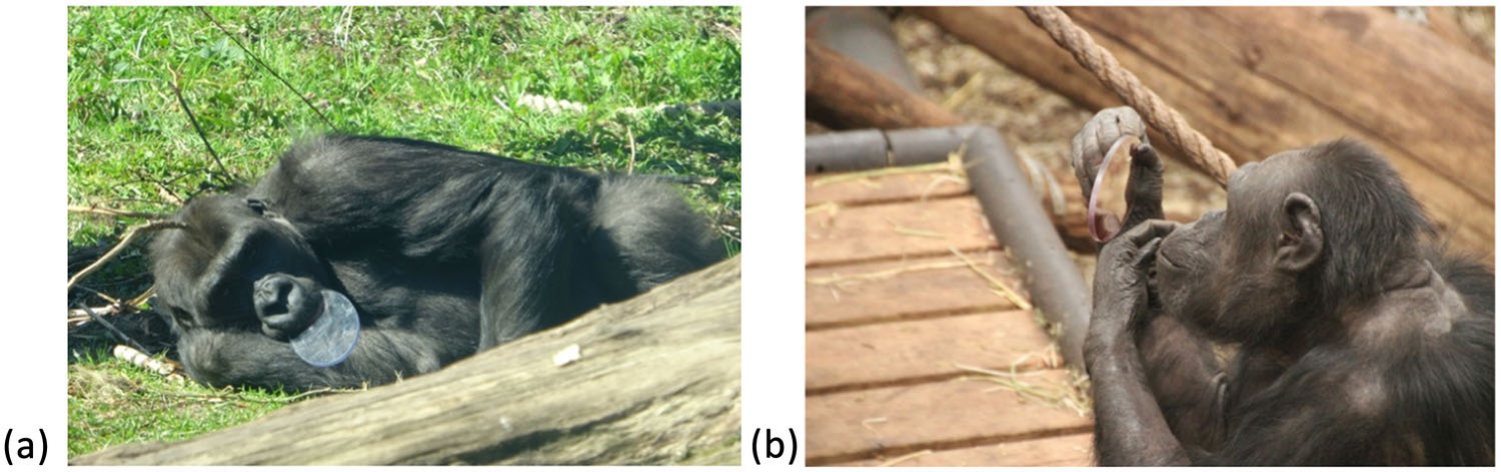
A gorilla manipulating (a) the control piece and (b) chimpanzee displaying self‐directed behaviors while holding the mirror.

The mirrors provided to the apes consisted of mirror‐foiled oval polycarbonate pieces (16 x 10 x 0.6 cm) with cleanly ground edges and orange foil on the back of the mirror. The control pieces consisted of plain transparent polycarbonates pieces of the same shape and dimensions (see Figure 1).

##### 2.3.1.2 Procedure

The experimental procedure followed was the same for both tested ape groups and lasted for four consecutive weeks. In the first and last week of the experiment, the apes were provided with the control pieces while they were given access to the mirrors in weeks 2 and 3. The respective number of objects provided was adapted to the group size and corresponded to the number of individuals in the group. The objects were distributed in the enclosure and left inside for five consecutive days, during which the individuals had ad libitum access to the objects. After 5 days, all objects were recollected, and the apes were given a 2‐day “experimental break” before providing the next set of objects. Observations were conducted from 7:30 to 15:30 in April and May of 2021 for the chimpanzee and gorilla groups respectively.

##### 2.3.1.3 Measures

We videotaped all interactions the apes had with the provided objects. The recordings were conducted with two stationary action cameras (Apexcam M80 Air) and one handheld camera (Sony FDR‐AX43). Videos were analyzed in Solomon Coder beta (Péter 2002), and coded behaviors were subsequently grouped into five categories (see Table S2 in the Supporting for ethogram): (1) Physical contact with the object reflecting active engagement with the objects (i.e., summed durations of all instances in which an individual was holding the object in its hand or mouth and transporting the object in its hand, mouth or on its back); (2) Close inspection (i.e., holding the object up close to the face at eye level, coded as a duration); (3) Object exploration (including slapping, biting, tapping, scratching, knocking, licking, sniffing and stepping on the object, coded as count measures); (4) Social behaviors exhibited while looking at the object (i.e., agonistic or affiliative behaviors and vocalizations, coded as count measures); (5) Self‐directed behaviors (i.e., touching their own face, ear, eye, auto‐grooming, and scratching) were measured when the individual was in close physical proximity to the object and particularly distinguished self‐directed behaviors performed while directly looking at the object (i.e., mirror‐guided self‐directed behaviors). An interrater reliability (i.e., ICC assessed by a two‐way model on the agreement between the raters) conducted on 10% of the video recordings (~16 h) between the raters RK and LV showed a high degree of reliability of 0.894 with 95% CI [0.875, high] (F = 17.9, *p* < 0.001).

It should be noted that the number of interactions analyzed for the gorillas is an underestimation of the number of interactions performed by the gorillas, as due to enclosure design, the gorillas had more areas out of sight for the experimenters where they may have interacted with the objects.

#### 2.3.2 Task 2: Body‐As‐Obstacle Task

##### 2.3.2.1 Material

**Figure 2:**
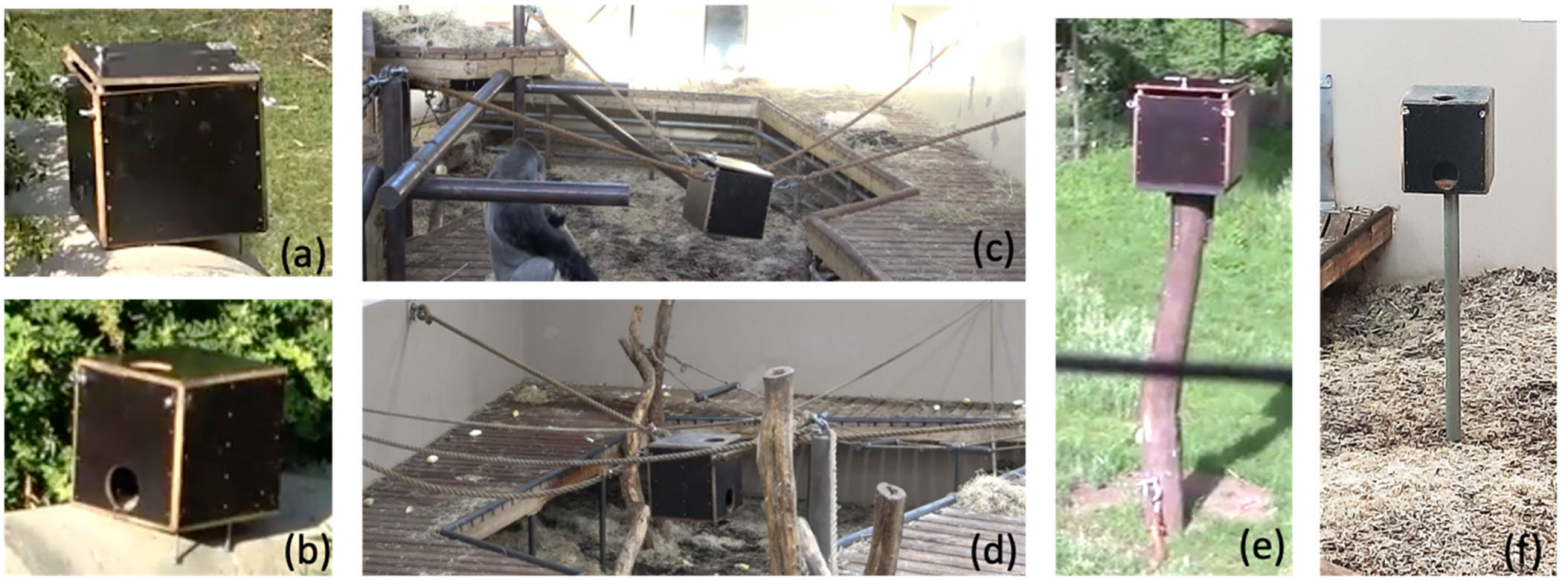
Apparatus used in the body‐as‐obstacle task depicting the test box (a, c, e) and control box (b, d, f) respectively in the ground (a, b), ropes (c, d) and pole (e, f) condition.

For this experiment, we exposed the apes to two types of wooden boxes (both 50 x 50 x 50 cm). Both boxes (Figure 2a,b) contained a commercial pet feeder (Pretty Paws PP005) that acted as a remote‐controlled food dispenser. One box (test box) had a top opening lid, which the apes had to lift to access the food dispensed by the feeder. The lid was fastened by a chain so that the apes had to actively hold the lid open to access the food, and the lid would automatically close if not held open. The test box was remotely rebaited by the experimenter when the lid closed. The second box (control box) had two round (15 cm diameter) openings, one on top and one on the side of the box enabling the apes to reach into the box to grab the dispensed food. The dispensing of food in the control box was set to dispense 40 portions of food at random intervals (of 1–10 min between dispenses) for 4 h or until the feeder was empty. The food dispensed by the feeder consisted of corn, seeds and/or biscuits made of corn and carrot, all (except the biscuits) part of the apes' everyday diet. Each food dispensing event released a small handful of food.

##### 2.3.2.2 Procedure

The apes were exposed to the box in a group setting and had ad libitum access to the box. The feeder was filled before each session and the observation period lasted until the feeder was depleted of food (or the 40 portions had been dispensed in the conditions with the control box).

The experiment started out with a habituation and training phase with the test box in which the box was fixed to the ground. The goal of the training was for the apes to get accustomed to opening the box to access a reward and to understand the need to close the lid before the box would get rebaited by the experimenter. The closing of the lid therefore marked the end of an ongoing trial. An individual was considered trained, when it had at least one successful trial i.e., when it closed the lid and waited for rebaiting, without exhibiting any other behaviors indicative of alternative food‐extraction attempts, like shaking the box, using a stick to access the feeder or remaining seated atop the box while holding the lid open after having consumed all the provided food. Training for the group ended after at least 80% of the individuals were considered trained on the box (i.e., 9 out of 11 gorillas had been observed interacting with the box successfully and 13 out of 15 chimpanzees). The apes further received a habituation period of 4 days in the Control box.

In the subsequent tests, both boxes were subsequently presented to the apes in three conditions. First, the box was presented to the apes to the ground, then suspended by ropes and finally mounted on a pole (Figure 2) for eight sessions each. The boxes remained in their location until the data collection for the eight sessions was completed. The apes were first exposed to the test box in all three conditions before being given access to the control box in the same conditions.

The ground condition for both boxes aimed at establishing baseline interaction behaviors with the boxes. The rope and pole conditions aimed to entice the apes to climb and sit on top of the box before they would attempt to extract food from the box (see video V3). Consequently, in the *Rope/Test Box* condition, the apes had the choice of sitting on the box in which case they would be required to move aside to be able to open the lid, as their own body (i.e., the weight of their body) presents an obstacle to the opening of the lid; or sit on the less stable ropes next to it (which presumably would require more effort than sitting on the flat and stable top of the box). Contrarily, in the *Rope/Control Box* condition, both options (sitting on top of the box) or next to it on the ropes were equally viable choices for the successful retrieval of the dispensed food, as they could use the hole on the top of the box to reach into the box when sitting on it. To successfully retrieve food from the box in the *Pole/Test Box* condition, the apes needed to climb on top of the box to be able to grasp the lid (thus becoming an obstacle to the opening of the lid) and then needed to step off by circumventing the lid while lifting it. Contrarily, in the *Pole/Control Box* condition, the apes could stay seated on top of the box while retrieving food through one of the holes and did not need to step off.

##### 2.3.2.3 Measures

We recorded all sessions with a handheld camera (Sony FDR‐AX4) and analyzed the footage in the behavioral coding software Loopy (© loopbio GmbH). Due to the group testing and the frequent presence of more than one individual at the box, we distinguished between *box users* (i.e., individuals who actively engaged with the box retrieving food) and *bystanders* (i.e., passive individuals who spent time close to the box, without manipulating it or retrieving food) and focused our analysis on the box users. In conditions involving the test box, we further differentiated between *opener* (i.e., the individual that pushed the lid open) and *non‐opener* (i.e., individuals that accessed the food in the box after another individual had opened it).

In all conditions, we measured the amount of time individuals spent actively using the box, and determined the position of the individual in relation to the box while using it (i.e., sitting next to the box, or on top of the box, further differentiating between sitting on the closed lid of the box or sitting on the box with an open lid in sessions with the test box) to calculate the respective proportions of time an individual spent sitting in each position by summing the time spent in each position for each individual and dividing it by the total amount of time the individual spent actively engaging with the box (i.e., retrieving food and waiting for food provisioning).

In the conditions involving the test box, we further measured the number of failed and successful opening attempts exhibited by the individual at the box (see Table S3 for a detailed ethogram of coded behaviors). Failed opening attempts consisted of instances in which the ape appeared to attempt to lift the lid while still standing on top of it or attempts to open the lid while another individual was sitting on the lid, blocking the opening. From these, we calculated two types of efficiency rates by dividing the number of successful attempts by the sum of successful and failed attempts. First, we calculated the individual's efficiency over all the trials performed by the individual. Second, we calculated an individual's efficiency in their first trial.

Videos for the BAO experiment were coded by two raters (AT and LV). An interrater reliability was conducted on ~12 h of video recordings of 4 separate days of data collection, amounting to ~4%. Raters had a high interrater reliability with an ICC = 0.99, F_(32,32.5)_ = 198, *p* < 0.001, 95% CI [0.979; 0.995].

### 2.4 Data Analysis

#### 2.4.1 General

All reported analyses were conducted in R (version 4.2.1, R Core Team 2020). To control for multiple demographic effects and repeated measures in our analyses, we ran several generalized mixed models on our data. All models were fitted using the generalized mixed model function of the glmmTMB package (version 1.1.7 (Brooks et al. 2017)). We checked for model convergence, overdispersion, and when part of the model assumptions, the normality of the random effects. We further ruled out collinearity issues by checking the variance inflation factors (“vif” function of the car package, version: 3.1‐0) obtained on a linear model lacking the random factor and the interaction term (Field 2005). All model comparisons were based on a likelihood ratio test performed with the “lrtest” function of the “lmtest” package (version 0.9‐40). Model diagnostics were checked visually as well as with the DHARMa package (Hartig 2022) (version 0.4.6) and performance package (version 0.10.3.4). Effects of individual model predictors were assessed using likelihood ratio tests comparing full models with their respective reduced models using the drop1 function with the test argument set to “Chisq.” Tukey adjusted post‐hoc pairwise comparisons were conducted using the emmeans package (version 1.7.5 (Lenth 2020)).

#### 2.4.2 Data Analysis of the Mirror Self‐Recognition Task

For behavioral categories comprised of behaviors coded as events, we added the number of all the exhibited behaviors included in this category per individual, condition and week. For behavioral categories comprise of duration behaviors, we added the absolute duration of the behaviors included in the category to get a summed duration variable per individual, condition and week for the behavioral category. To analyze the absolute duration of *close inspection* and of *physical contact* with the object, we fitted two generalized linear mixed‐effect models with beta distribution and a logit link function, and we kept the phi constant on rescaled data. The data were rescaled to be between 0 and 1 (and compressed to exclude zeros and ones) according to the following formula: *y*_i_´= (*y*_i_‐*y*_min_)/(*y*_max_ – *y*_min_) *((*n*‐1) + 0.5)/*n*) with n equal to the sample size (Smithson and Verkuilen 2006). In these models, we included condition (control/mirror), species (chimpanzee/gorilla), age, block (week1/week2 corresponding to the first and second week of testing in each condition) and the interaction term between species and condition as fixed factors, and the individual's identity as a random factor. To assess the inclusion of the interaction term, we compared both full models to reduced models (identical to the full model including the main effect terms included in the interaction but lacking the interaction term) and to null models lacking all fixed factors included in the full model but retaining the random effect. For the physical contact variable both the full (*df* = 5, *χ* ^2^ = 34.241, *p* < 0.001) and reduced (*df* = 4, *χ* ^2^ = 32.953, *p* < 0.001) model were significantly better than the null model, however, the inclusion of the interaction term did not improve the model (*df* = 1, *χ* ^2^ = 1.288, *p* = 0.256). Similarly, for the close inspection variable, both the full (*df* = 5, *χ* ^2^ = 15.648, *p* = 0.008) and reduced (*df *= 4, *χ* ^2^ = 11.843, *p* = 0.018) model were significantly better than the null model. However, the inclusion of the interaction term did not improve the model (*df* = 1, *χ* ^2^ = 3.805, *p* = 0.051). Consequently, we excluded the interaction term and retained the reduced model for further analysis (Engqvist 2005).

To analyze the absolute frequency of *exploration behaviors* exhibited by the individuals towards the objects, we fitted a generalized linear mixed‐effect model with a negative binomial (nbinom2) distribution including condition (control/mirror), species (chimpanzee/gorilla), age, order (block1/block2) and the interaction term between species and condition as fixed factors, and the individual´s identity as a random factor. We compared the full model to reduced models (that lacked the interaction term but included the terms of the interaction as main effects) and a null model lacking all fixed factors included in the full model but retaining the random effect. The results showed that the full (*df* = 5, *χ* ^2^ = 57.966, *p* < 0.001) and the reduced model (*df* = 4, *χ* ^2^ = 38.994, *p* < 0.001) were significantly better than the null model and that the inclusion of the interaction term significantly improved the model (*df* = 1, *χ* ^2^ = 18.973, *p* < 0.001), we therefore kept the full model.

Due to a very low occurrence rate of *social behaviors*, *self‐directed behaviors and mirror guided self‐directed*, we did not fit models but reported the number of occurrences and number of individuals exhibiting these behaviors for each species and condition (see Table S5).

#### 2.4.3 Data Analysis for the Body‐as‐Obstacle Task

As previously mentioned, due to the passing of one individual during the study and the illness of another individual for parts of the study, these individuals were excluded from all statistical analyses that included the data from the control box condition. We further had to exclude two recordings due to data corruption resulting in parts of the data for the chimpanzees' fourth day of data collection for the test box in the ground condition and the gorillas' first day of the test box in the ropes condition missing from the analyzed dataset.

##### 2.4.3.1 Box Opening Efficiency

We assessed two measures of efficiency in the opening of the test‐box, first over all trials in which the individual participated and second examining the attempts (number of failed attempts until successfully opening the box for the first time. For both variables, we fitted a generalized linear model with beta distribution and logit link function with the phi kept constant, which included the species (Chimpanzee/Gorilla), the condition (Ground/Rope/Pole) and the age of the individual as fixed factors and individual as a random factor. The model was fitted to rescaled data. The data were rescaled to fit a 0 <y < 1 interval according to the following formula: *y*_i_´= *y*_i_*((*n*‐1) + 0.5)/*n*) with *n* equal to the sample size. As a test of the effects of the fixed factors included in our model, we conducted a comparison between our full model and a null model lacking the fixed effects but retaining the random effect included in the full model.

##### 2.4.3.2 Position Preferences

We first examined the amount of time individuals spent on top of the box. We here included the data of all box users (i.e., individuals who in that trial retrieved food from the box). We rescaled the data following the *y*_i_´ = *y*_i_*((*n*‐1) + 0.5)/*n*) formula. We fitted a generalized linear model with a beta distribution for which we kept the phi constant. The model included the condition (ground/ropes/pole), the box type (control/test), the interaction between the box and condition, the species (chimpanzee/gorilla) and the age of the individuals as fixed factors and Individual was included as a random factor. We compared the full model to a reduced model (that lacked the interaction term but included the terms of the interaction as main effects) and a null model lacking all fixed factors included in the full model but retaining the random effect. The results showed that the inclusion of the interaction term significantly improved the model (full‐reduced model comparison: *df* = 2, *χ* ^2^ = 28.849, *p* < 0.001); we therefore kept the full model.

We further explored the proportion of time the individuals spent on top of the lid in the test box sessions only and similarly fitted a generalized linear model with beta distribution to the rescaled data. The model included the condition (ground/ropes/pole), the species (chimpanzee/gorilla) and the age of the individual as fixed effects and the individual as a random effect.

#### 2.4.4 Comparison Between the Two Self‐Awareness Tasks

To compare the performances of the individuals in both tasks we assessed the correlation (Pearson´s correlation coefficient) between the amount of mirror‐guided self‐directed behaviors performed in the mirror condition of the MSR task and the efficiency in the pole condition of the BAO task.

## 3 Results

### 3.1 Mirror Self‐Recognition Task

#### 3.1.1 Physical Contact

**Figure 3:**
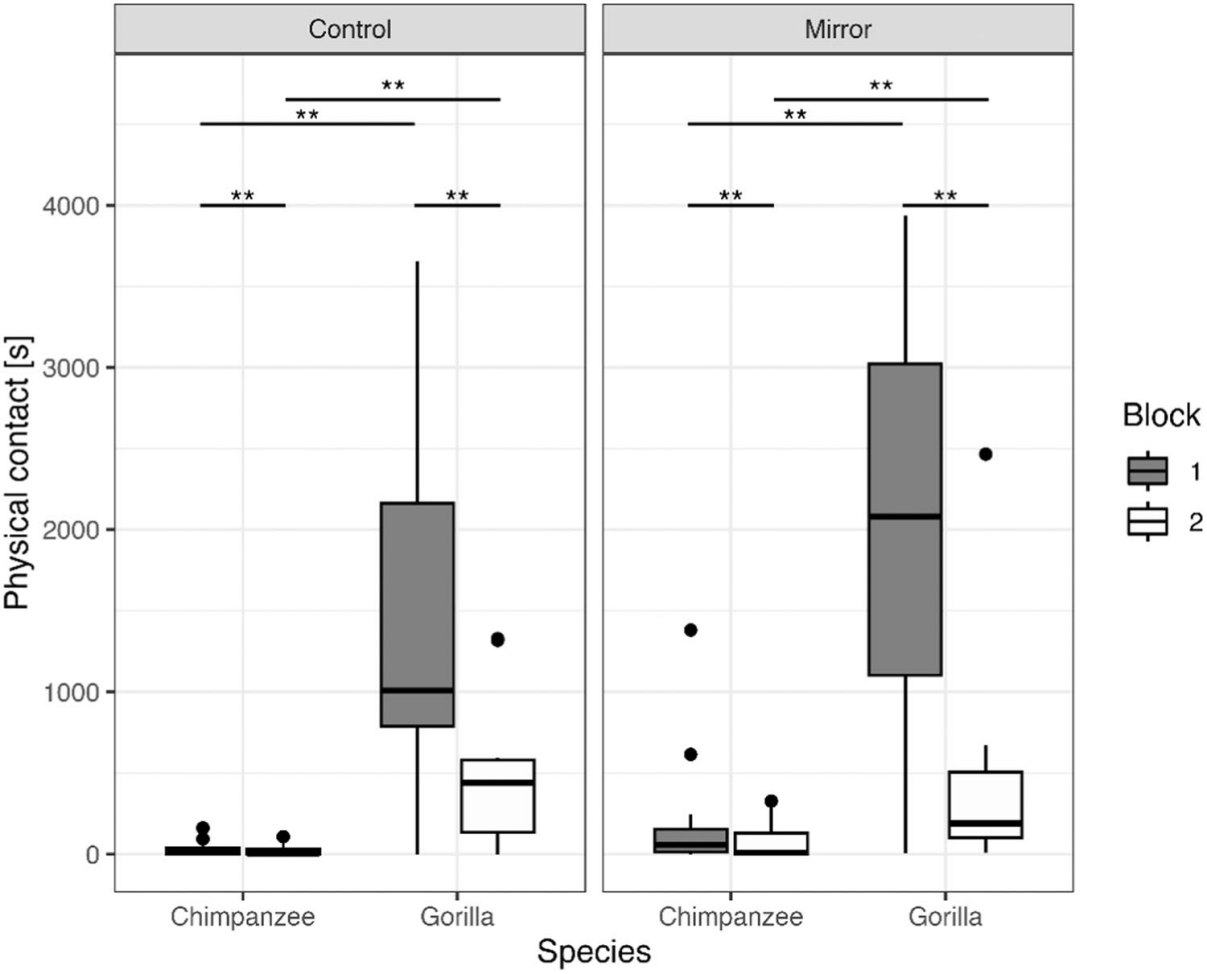
Summed duration of physical contact (in seconds) with the mirror or the control Plexiglas object by the chimpanzees and gorillas in the first and second week of exposure (i.e., blocks 1 and 2). Box‐plot lines represent medians, boxes represent the upper‐ and lower quartile range, whiskers represent ±1.5x the respective interquartile range, full back points represent outliers.

Overall, we recorded 14/15 chimpanzees and 10/11 gorillas interacting with the objects provided in this experiment for a total of 4919 s (min = 0, max = 1381.4 s, Median (IQR) = 14.1 s (76.2)) and 43818 s (min = 0, max = 3936.4 s, Median (IQR) = 631.8 s (1363.25)) respectively. We found that the factors in our model had an effect on the amount of time individuals spend in physical contact with the objects (full‐null model comparison: *df* = 4, *χ* ^2^ = 32.953, *p* < 0.001). We found that only species (*p* < 0.001) and test block (*p* < 0.001) but not the condition (*p* = 0.722) nor the age (*p* = 0.980) had an influence on the amount of time individuals spent interacting with the object. Overall, both species showed a decrease in the amount of interaction they had with both types of objects between block 1 and block 2 (Est. = 0.783, 95% CI [0.214, 1.351], Chimpanzee: *p* = 0.004; Gorilla: *p* = 0.004). And although neither species interacted significantly more with either one of the two types of objects presented to them (control or mirror), the gorillas spent overall more time interacting with the objects than the chimpanzees in both blocks (Chimpanzee vs. Gorilla: Est. = −1.735, 95% CI [−3.005, 0.464], Control_block1_: *p* = 0.009; Mirror_block1_: *p* = 0.009; Control_block2_: *p* = 0.009; Mirror_block2_: *p* = 0.009) (Table 1; Figure 3).

**Table 1:** Model results for physical contact, close inspection (estimates, standard error, 95% confidence interval, z values and p‐value).

*Predictors*	Physical contact					Close inspection				
	*Estimates*	*SE*	*CI*	*z*	*p*	*Estimates*	*SE*	*CI*	*z*	*p*
(Intercept)	−2.264	0.599	–3.439, –1.089	–3.777	< 0.001	–1.640	0.555	–2.728, –0.551	–2.953	0.003
Species [Gorilla]	1.168	0.456	0.725, 2.512	3.551	< 0.001	0.018	0.415	–0.794, 0.831	0.044	0.964
Condition [Mirror]	0.067	0.188	−0.302, 0.436	0.356	0.722	0.473	0.232	0.035, 0.910	2.118	0.034
Age	0.004	0.015	−0.029, 0.031	0.025	0.980	–0.015	0.015	–0.044, 0.014	–1.041	0.298
block [2]	−0.793	0.216	–1.217, –0.369	–3.666	< 0.001	–0.628	0.230	–1.079, –0.176	–2.725	0.006

#### 3.1.2 Close Inspection

The duration spent closely inspecting the object (i.e., holding the object closely up to their face on eye level) was affected by the factors included in our model (full‐null model comparison: *df* = 4, *χ* ^2^ = 11.843, *p* = 0.019). Time spent in close inspection was significantly affected by the block (*p* = 0.014) and showed a trend towards a condition effect (*p* = 0.071, both species tended to show more close inspection towards the mirror than the control). Specifically, we found that both species exhibited significantly more close inspection behaviors in the first exposure week (i.e., block 1) than in the second week (Table 1). The duration of close inspection was however not affected by the species (*p* = 0.964) or the age of the individual (*p* = 0.295).

#### 3.1.3 Exploration Behaviors

**Figure 4:**
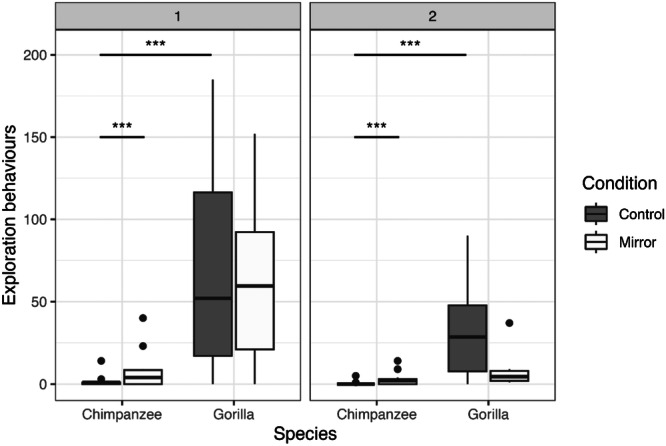
Number of explorative behaviors directed towards the mirror and the control Plexiglas object by the chimpanzees and gorillas in the first and second week of exposure (i.e., blocks 1 and 2). Box‐plot lines represent medians, boxes represent the upper‐ and lower quartile range, whiskers represent ±1.5x the respective interquartile range, full back points represent outliers.

Overall, we found that the factors in our model influenced the exhibition of exploration behaviors (full‐null model comparison: *df* = 5, *χ* ^2^ = 57.966, *p* < 0.001). The test block (*p* < 0.001) and the interaction between species and condition (*p* < 0.001) had significant effects on the amount of exploration behaviors directed towards the objects (Table 2; Figure 4). More specifically, we find that both species exhibited significantly more exploration behaviors towards both objects in the first week of exposure than in the second week (block1 vs. block2: Est. = 1.21, 95% CI [0.452, 1.963], Chimpanzee_Control_: *p* < 0.001; Chimpanzee_Mirror_: *p* < 0.001; Gorilla_Control_: *p* < 0.001; Gorilla_Mirror_: *p* < 0.001; Est. = 1.21, 95% CI [0.452, 1.963]). The gorillas in both blocks exhibited significantly more exploration behaviors towards the control object than the chimpanzee, yet there was no difference between species regarding the exploration behaviors exhibited towards the mirror (Chimpanzee vs. Gorilla in Control_block1_: Est. = −4.08, 95% CI [−6.575, −1.581], *p* < 0.001; Control_block2_: Est. = −4.08, 95% CI [−6.575, −1.581], *p* < 0.001; Est. = −4.08, 95% CI [−6.575, −1.581], Mirror_block1_: Est. = ‐1.51, 95% CI [−3.814, 0.784], *p* = 0.752; Mirror_block2_: Est. = −1.51, 95% CI [−3.814, 0.784], *p* = 0.752). Finally, the chimpanzees explored the objects significantly more in the mirror condition, yet we found no condition effect in the gorillas (Control vs. Mirror: Chimpanzee: *p* < 0.001; Chimpanzee_block2_: Est. = −1.852, 95% CI [−2.828, −0.876], *p* < 0.001; Gorilla: Est. = 0.712, 95% CI [−0.209, 1.633], *p* = 0.803) (Figure 4).

**Table 2:** Model results exploration behaviors (estimates, standard error, 95% confidence interval, z values and p‐value).

*Predictors*	*Estimates*	*SE*	*CI*	*z*	*p*
(Intercept)	0.854	1.134	−1.368, 3.076	0.753	0.451
Species [Gorilla]	3.787	0.911	2.001, 5.572	4.156	< 0.001
Condition [Mirror]	1.879	0.387	1.120, 2.638	4.850	< 0.001
Age	−0.036	0.030	−0.095, 0.022	−1.227	.220
block [2]	−1.165	0.249	−1.653, −0.677	−4.682	< 0.001
Species [Gorilla]:Condition [Mirror]	−2.413	0.529	−3.450, −1.376	−4.562	< 0.001

#### 3.1.4 Social Behaviors

We recorded a total of 10 exhibitions of social behaviors by four individuals (three chimpanzees and one gorilla) (Table S5). The chimpanzees exhibited more social behaviors in both conditions than the gorillas (in the control condition, chimpanzee: M = 0.667, SD = 0.254 vs. gorillas: M = 0.10, SD = 0.447; in the mirror condition, chimpanzee: M = 0.233, SD = 0.971 vs. gorillas: M = 0, SD = 0).

#### 3.1.5 Self‐Directed Behaviors

We observed a total of 81 instances of self‐directed behaviors performed while the individual was in close proximity to an object (chimpanzees: min = 0, max = 26, Median (IQR) = 0 (0.75); gorillas: min = 0, max = 11, Median (IQR) = 0 (2.75). Forty‐one instances were exhibited by chimpanzees (*n* = 7) in the mirror condition, 23 by gorillas (*n* = 5) in the mirror condition, 1 by a chimpanzee in the control condition and 16 by gorillas (*n* = 4) in the control condition. Out of these 81 instances, only 27 were mirror‐guided (i.e., exhibited while the individual was looking at the object). 26/27 of these events were performed by two chimpanzees (Erika: 22 instances, Tushi: 4 instances) in the mirror condition and 1 event was exhibited by a gorilla in the mirror condition as well (Table S5 and videos V1 and V2 of the Supporting material for examples of mirror‐guided self‐directed behaviors in the chimpanzees and gorilla).

### 3.2 Body‐as‐Obstacle Task

In the body as obstacle task, we observed all individuals approaching the box. They spent a total of 662536.2 s in close proximity to the box (i.e., next to, or on top of the box or lid) of which the chimpanzees spent a total of 426446.1 s (min = 2047.2 s, max = 71945.16, M = 28429.04, SD = 21091.40) and the gorillas a total of 236090.2 s (min = 376.96, max = 51237.56, M = 21462.74, SD = 17613.76) at the box. All individuals opened the box and retrieve food from the box at least once (number of box openings by chimpanzees: M = 119.07, SD = 105.73, min = 1, max = 309, and by gorillas: M = 155.28, SD = 133.51, min = 3, max = 435).

#### 3.2.1 Box Opening Efficiency

**Figure 5:**
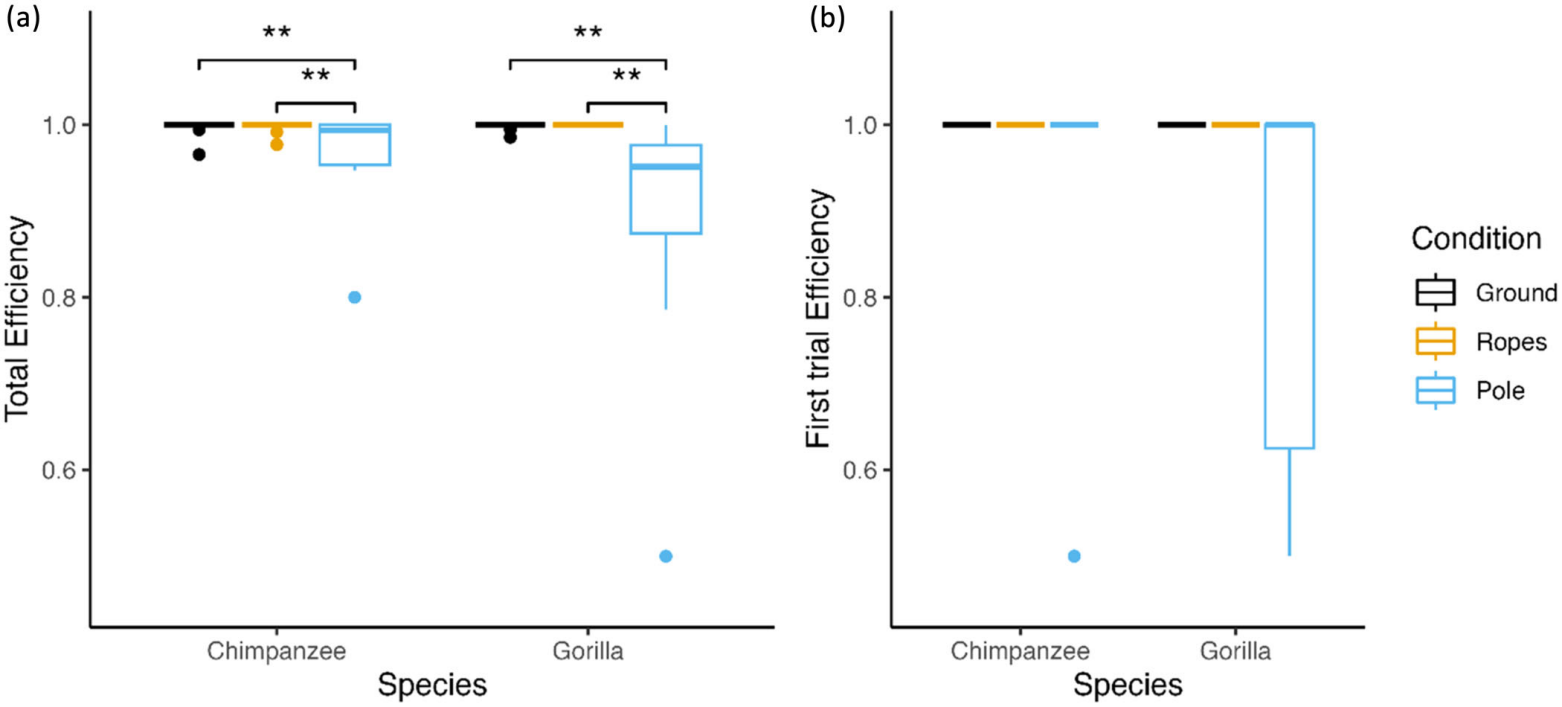
Test box opening efficiency of the chimpanzees and gorillas in the three test conditions (with the box on the ground, on the ropes and on the pole) (a) overall trial and (b) on their first attempt only. Box‐plot lines represent medians, boxes represent the upper‐ and lower quartile range, whiskers represent ±1.5x the respective interquartile range, full back points represent outliers.

Overall, we found that the factors in our model had an effect on the total efficiency (full‐null model comparison: *df* = 4, *χ* ^2^ = 15.268, *p* = 0.004). Across all trials, the box opening efficiency of the tested individuals was significantly influenced by the test condition (*p* < 0.001) but not the species or the age of the individual. The individuals made significantly more failed attempts in the pole condition than in the other two conditions (Ground vs. Pole: Est. = 0.866, 95% CI [0.288, 1.444], *p* = 0.003; Ropes vs. Pole: Est. = −0.042, 95% CI [−0.646, 0.562], *p* = 0.003; Ground vs. Ropes: Est. = −0.908, 95% CI [−1.501, −0.315], *p* = 0.994, Table 3; Figure 5a). Yet, the performances in the first trial were not affected by any of the factors in the model (full‐null model comparison: *df* = 4, *χ* ^2^ = 2.747, *p* = 0.601, Figure 5b).

**Table 3:** Model results for the box opening efficiency over all trial (Total efficiency) and the duration spent on the lid of the box, of individual in each of the three test conditions (estimates, standard error, 95% confidence interval, z values and p‐value).

*Predictors*	Total Efficiency					Time on the lid				
	*Estimates*	*SE*	*CI*	*z*	*p*	*Estimates*	*SE*	*CI*	*z*	*p*
(Intercept)	3.545	0.465	2.665, 4.488	7.624	< 0.001	−3.191	0.598	−4.364, 2.019	−5.337	< 0.001
Species [Gorilla]	−0.047	0.307	−0.722, 0.458	−0.152	0.879	0.0458	0.400	−0.737, 0.829	0.115	0.909
Age	0.012	0.012	−0.013, 0.034	0.981	0.327	−0.023	0.016	−0.054, 0.009	−1.421	0.155
Condition [Pole]	−0.813	0.250	−1.349, −0.382	−3.245	0.001	1.046	0.216	0.623, 1.469	4.848	< 0.001
Condition [Ropes]	0.027	0.261	−0.463, 0.547	0.102	0.919	0.597	0.222	0.161, 1.033	2.684	0.007

#### 3.2.2 Position Preferences

**Figure 6:**
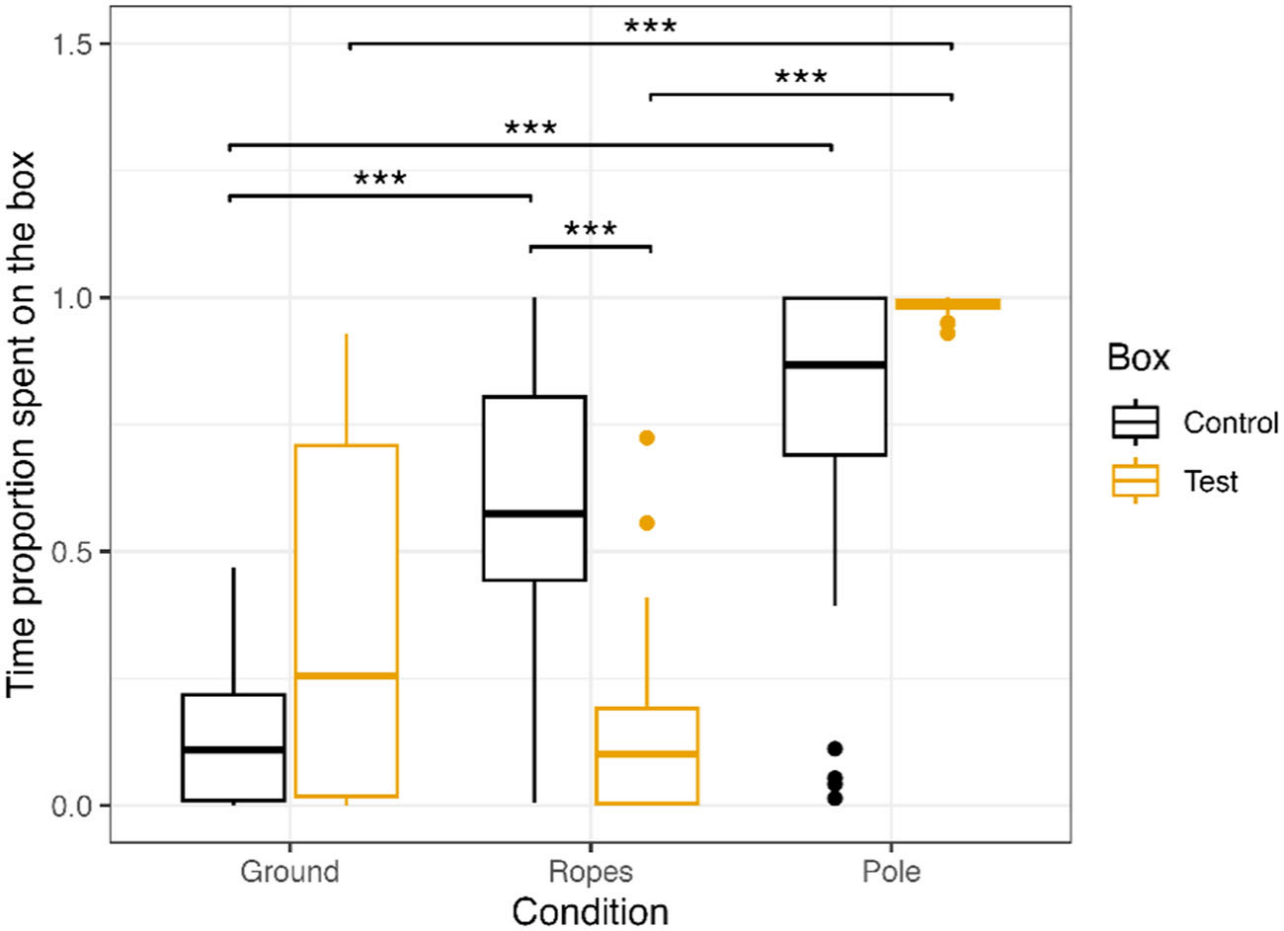
Proportion of time spent sitting on top of the test and control box in all three conditions (box positioned on the ground, suspended on ropes and on a pole). Box‐plot lines represent medians, boxes represent the upper‐ and lower quartile range, whiskers represent ±1.5x the respective interquartile range, full back points represent outliers.

For the proportion of time the individuals spent on the box (full‐null model comparison: *df* = 7, *χ* ^2^ = 93.464, *p* < 0.001), we found that the age of the individual (*p* = 0.009) and the interaction factor between box and condition (*p* < 0.001) had a significant effect on the amount of time the individual spent sitting on the box rather than next to the box (Table 4). Older individuals (particularly in the gorillas) spent less time on the box than younger individuals. Post‐hoc pairwise comparisons on the effects of the box and condition revealed that the individuals spent significantly less time on the control box in the ground condition than in the rope (Est. = −1.669, 95% CI [−2.690, −0.647], *p* < 0.001) or pole condition (Est. = −2.271, 95% CI [−3.313, −1.230], *p* < 0.001). The individuals spent significantly less time on the test box in the ground and rope condition than the pole condition (Ground vs. Pole: Est. = −2.541, 95% CI [−3.587, −1.496], *p* < 0.001; Rope vs. Pole: Est. = −3.083, 95% CI [−4.130, −2.036], *p* < 0.001). Individuals further spent significantly more time on the control than the test box in the ropes condition (Est. = 1.620, 95% CI [0.654, 2.586], *p* < 0.001) but not in the pole (Est. = −0.860, 95% CI [−1.841, 0.120], *p* = 0.124) nor in the ground (Est. = −0.591, 95% CI [−1.586, 0.405], *p* = 0.538) condition (Figure 6).

**Table 4:** Model results of proportion of time spent on the box (estimates, standard error, 95% confidence interval, z values and p‐value).

*Predictors*	Time spent on the box				
	*Estimates*	*SE*	*CI*	z	*p*
(Intercept)	−0.284	0.489	−1.243, 0.674	−0.581	0.561
Species [Gorilla]	−0.525	0.309	−1.131, 0.080	−1.700	0.089
Box [Test]	0.591	0.349	−0.094, 1.275	1.691	0.091
Condition [Ropes]	1.669	0.358	0.966, 2.371	4.656	< 0.001
Condition [Pole]	2.271	0.365	1.555, 2.988	6.216	< 0.001
Age	−0.034	0.013	−0.059, −0.008	−2.620	0.009
Condition [Ropes]: Box [Test]	−2.211	0.492	−3.175, −1.246	−4.494	< 0.001
Condition [Pole]: Box [Test]	0.270	0.490	−0.690, 1.229	0.551	0.582

We further examined the proportion of time individuals spent on the closed lid of the test box (full‐null model comparison: *df* = 4, *χ* ^2^ = 26.132, *p* < 0.001) which showed a significant effect of the condition (*p* < 0.001) but not of species (*p* = 0.909) nor of the age (*p* = 0.152) of the individual (Table 3). The individuals spent significantly more time sitting on the lid of the test box in the pole (Est. = −1.046, 95% CI [−1.552, −0.540], *p* < 0.001) and rope (Est. = −0.597, 95% CI [−1.118, −0.076], *p* = 0.020) condition than in the ground condition, and a trend for spending more time on the lid in the pole condition than the rope condition (Est. = 0.449, 95% CI [−0.008, 0.905], *p* = 0.055).

### 3.3 Task Performance Comparisons

**Figure 7:**
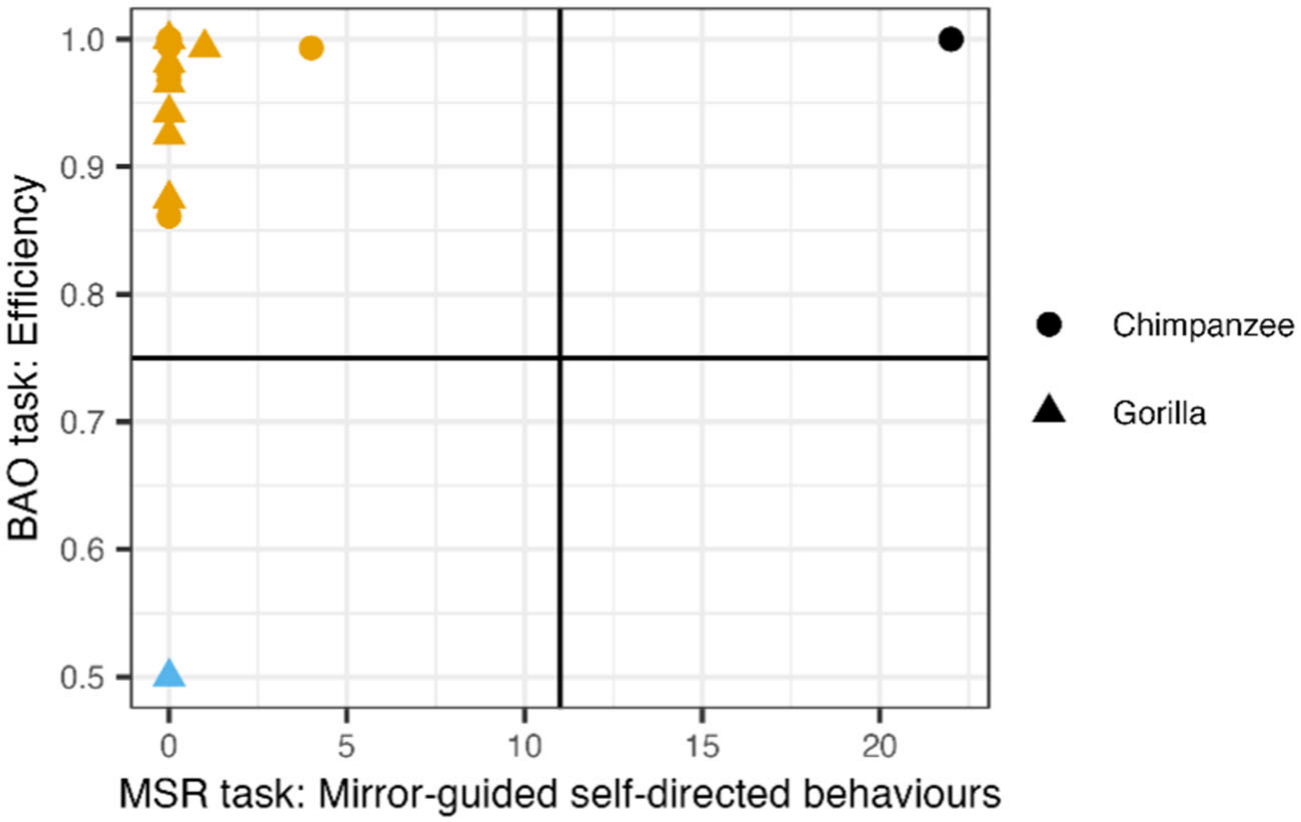
Correlation plot of task performance with the results for the mirror self‐recognition (MSR) task (on the x‐axis) i.e., number of mirror‐guided self‐directed behaviors exhibited towards the mirror, and the results for the body‐as‐obstacle task (BAO) i.e., efficiency in opening the test box in the pole condition (on the y‐axis). Horizontal and vertical lines represent the mean between the highest and lowest value of the variable measured and colors code for the quadrants.

We explored the task performances of the individuals between the two tasks and found no correlation between the average efficiency in opening the test box of the BAO task in the pole condition and the exhibition of mirror‐guided self‐directed behaviors in the MSR task (*t* = 0.542, *df* = 19, *p* = 0.594, *R* = 0.123; Figure 7).

## 4 Discussion

The present study investigated the performance of chimpanzees and gorillas in two self‐awareness‐related tasks, i.e., a mirror self‐recognition task (employing small handheld mirrors) and a BAO task, to explore whether, like human infants, the performances of great apes in these two tasks correlate. Comparing chimpanzees and gorillas in this study, we found that both species performed overall similarly in both tasks presented to them. In the mirror self‐recognition task, both species showed similar levels of interaction (i.e., holding, touching, transporting) in both conditions and did not interact more with the mirror than with the control object as we might have expected, but the gorillas showed significantly more interest in the objects presented to them than the chimpanzees overall. The test condition did, however, affect the behaviors the individuals exhibited while interacting with the object. Both species showed a tendency to spend more time exhibiting close inspection behaviors of the mirror and the chimpanzees, contrary to the gorillas, also exhibited more exploration behaviors towards the mirror than the control object. Yet only two chimpanzees exhibited clear signs of mirror self‐recognition by exhibiting mirror‐guided self‐directed behaviors while looking into the mirror. In the BAO task, both species spontaneously solved the BAO task and exhibited overall high‐efficiency rates in all conditions, even if both species showed more errors in the pole condition. We could further show that the individuals spent more time on the control box than on the test box in the rope condition (when both positions sitting next to the box and sitting on the box were expected to grant equal access to the food in the box), which might indicate that the apes actively avoided sitting on the test box, to avoid creating an obstacle to the box opening with their own bodies. Finally, we did not find a correlation between the individuals' performances in the two self‐awareness tasks presented to them in this study.

### 4.1 Mirror Self‐Recognition Task

#### 4.1.1 Habituation Effect

Although the gorillas did overall show more interest in the objects presented to them in the experiment than the chimpanzees, we observed a quick drop in interest in both objects for both species (see Figure S1 in the Supporting material), which is an effect often described in great apes in their interactions with mirrors (Anderson and Roeder 1989; Povinelli et al. 1993a; Suarez and Gallup 1981; Walraven et al. 1995) and objects in general (Paquette and Prescott 1988; Welker 1956). This effect could have been amplified in our setup due to the constant access to the objects throughout the week.

#### 4.1.2 Species Differences

On a species level, we observed rather low interest rates in the chimpanzees in this study and found that the gorillas interacted more with the objects than the chimpanzees. We expected such an effect due to the higher proportion of elderly individuals in the chimpanzee group and the presence of sub‐adults in the gorilla group as elderly individuals tend to engage in less object manipulation (Baker 2000) than younger individuals that tend to be more curious and explorative. However, contrarily to our expectations the age of the individuals did not have a significant effect on our behavioral measures, suggesting that the differences should be attributed to the groups and/or individual preferences rather than the subjects' age. This is in line with the studies from Povinelli and colleagues (1993), who found that adult chimpanzees did not spend less time looking at mirrors than younger individuals (however, there were no data available on individuals past the age of 40 in that study).

In line with previous findings, we observed that the gorillas, like the chimpanzees, exhibited pronounced interest in the objects and their own reflections (Posada and Colell 2007; Shillito et al. 1999; Suarez and Gallup 1981), holding the mirror up to their face in front of their eyes (i.e., close inspection), confirming the findings of Shillito and colleagues (1999) that gorillas do not seem to show gaze aversion towards the mirrors. The avoidance of eye contact, thus, does not appear to have been a hindering factor to the expression of MSR in gorillas in this study. Although the direct line of sight could not be determined, it would appear surprising that any individual would hold up a mirror to their eyes to not look at it. It is further noteworthy, that we observed close to no social behaviors when interacting with the mirror, the few observed social behaviors mostly consisted of vocalizations emitted while holding the object and were not necessarily directed at the object itself. This appears in line with the hypothesis of Kopp and colleagues (2021) that the use of small mirrors reduces the potential for agonistic behaviors towards mirror reflection. However, future studies would benefit from directly comparing the reactions of gorillas to large and small mirrors to establish whether this observation can be accredited to the object type, as seen in chimpanzees (Kopp et al. 2021).

Studies investigating the mirror self‐recognition abilities of gorillas chronically suffer from low sample sizes and inconsistent findings (see Murray et al. 2022 for review). Yet, despite a reasonable sample size, we found that none of the 11 gorillas tested in this study showed convincing signs of mirror self‐recognition. A finding that is in line with the current literature on self‐recognition in gorillas and cannot, as mentioned above, be explained by a lack of interest in the object's gaze aversion, nor even a lower rate of self‐directed behaviors exhibited in close proximity to a mirror. Although we observed a brief instance of partially mirror‐guided self‐directed behavior in one gorilla (video V2 of the Supporting material), the brief nature of this event and the lack of subsequent occurrences led us to adopt the more conservative conclusion that this single event was insufficient to demonstrate mirror self‐recognition.

Contrary to the gorillas, at least two of the chimpanzees exhibited signs of mirror‐guided self‐directed behaviors. Although it is well‐established that chimpanzees are capable of recognizing themselves and perform self‐directed behaviors in the presence of mirrors, studies report considerable interindividual variation in performance (Povinelli et al. 1993a). For instance, Swartz and Evans (1991) reported in their study that only 1 out of 11 chimpanzees passed the mark test, while Povinelli and colleagues (1993) reported that 30 out of 92 chimpanzees exhibited at least some self‐directed behaviors. Recent studies have suggested that these interindividual variations could be the result of differences in the individuals' genotypes or neuroanatomy (Hecht et al. 2017; Hopkins et al. 2019; Mahovetz et al. 2016). Besides these factors, age is an essential predictor of chimpanzees' performance in a MSR task, but not of the mirror viewing time (Povinelli et al. 1993b). About 80% of chimpanzees between the ages of 8–16 years of age show signs of self‐recognition, yet this percentage falls to 35% in chimpanzees in higher age ranges (de Veer et al. 2003; Povinelli et al. 1993b). However, with a success rate of 13%, this study lies below expectations, even with the age of the individuals taken into consideration. Anecdotally, it might be interesting to note, that the female that was the most prolific in her mirror‐guided self‐directed behaviors (Erika) used the mirror to inspect a small abscess she had on her nose at the time of the experiment and to observe her attempts to drain it. Although self‐directed behaviors exhibited toward a mirror are a good predictor for an individual's ability to recognize itself and pass a subsequent mark test (Povinelli et al. 1993a), the case of Erika shows that a supplementary (external) motivation seemed to enhance the exhibition of self‐directed behaviors and that the implementation of a mark test would have been useful in this context for a clearer assessment of the individuals mirror self‐recognition abilities.

Ultimately, despite not drastically differing in their interaction with the mirrors, only the chimpanzees exhibited some instances of behaviors indicative of mirror self‐recognition, reinforcing the idea that gorillas generally do not exhibit or possess MSR, either due to a secondary loss of this ability during evolution or a very low expression rate of this ability, if MSR is considered a polymorphic trait within a population.

### 4.2 Body‐as‐Obstacle Task

Overall, both the chimpanzees and gorillas performed very well in the BAO task presented to them in this study. Although both species made significantly more mistakes in the pole condition, they still had very high success rates (efficiency_Chimpanzee_ M = 0.983, SD = 0.037 and efficiency_Gorilla_ M = 0.967, SD = 0.124) in the pole condition (Table S4), surpassing those observed in 21‐month‐old children reported by Moore and colleagues (2007) in the shopping cart task. Moore and colleagues (2007) concluded that, at this age, the children seemed to solve the BAO task more by trial and error than through true insight (Moore et al. 2007). Taking the first trial efficiency and the total efficiency of the apes into account, the great apes were able to solve the task spontaneously, including the pole condition in which their bodies presented an obstacle to the completion of the task. These results might suggest that the chimpanzees and gorillas did not only understand the task at hand but did so using insight for their problem‐solving. This interpretation is further supported by the finding that the apes appeared to actively avoid sitting on top of the test box in the ropes condition, thus avoiding a sitting position in which their bodies could get in the way of the action they wanted to perform, despite the less stable seating position. Nevertheless, we cannot exclude that the high success rates overall, and in the pole condition in particular, were also the result of some learning that took place in the conditions before the pole condition, or through social learning by observing successful individuals, as the apes, contrarily to the infants, were tested in groups and not individually. We further cannot exclude the role played by the sensory feedback of the lid movement on the body and its effects on the individuals' behaviors, which could have motivated the individuals to step off the lid.

It thus appears that both ape species, despite the gorillas being considered more terrestrial and performing worse in mirror self‐recognition tasks than the chimpanzees, understood that their bodies were obstacles to the action they wanted to perform (i.e., opening the lid to access the food) which would, according to current theories, suggest that they possess some level of BSA, or at least a sense of agency and possibly a sense of body ownership, including a mental representation of their own body. Further evidence that the apes might possess a body image and a sense of body ownership (De Vignemont 2010; Gallagher 1986, 2000) could come from instances of behaviors observed in the control box sessions of the BAO task, in which the chimpanzees and gorillas were observed looking through the top hole while inserting their hands through the side hole of the box to guide their food retrieval. As this action creates a discontinuity between the hand and the rest of the body, these behaviors might be indicative of a sense of body continuity and a mental representation of the body as a whole. Yet, whether this reflects a conscious form of BSA and body ownership is still at issue (Blanke and Metzinger 2009).

### 4.3 Comparing Paradigms

In the final part of this study, we examined whether, similarly to human infants, we could find a correlation between the ability to solve the mirror self‐recognition task and the ability to solve a BAO task in great apes, to explore whether these two abilities might draw from the same underlying ability of objective self‐awareness. Yet, contrarily to our expectations and the findings in children (Moore et al. 2007), we did not find a correlation between the performances in the MSR and BAO tasks in the chimpanzees and gorillas; i.e., individuals that exhibited signs of MSR did not perform better (or worse) in the BAO task than the other individuals. However, this result needs to be interpreted with the appropriate level of caution, considering the very low success rate (and resulting floor effect) in the MSR task, leading to a small sample size (n = 2) for the group of self‐recognizing individuals and the ceiling effect in the BAO task. The resulting narrow range of variation could have made detecting interindividual differences more difficult and could therefore have biased the correlation results, leading to a potential underestimation of the correlation. Future investigations might, therefore, benefit from implementing a more difficult BAO task to achieve a wider dispersion, allowing for a more accurate assessment of the correlation between the two tasks.

However, presuming that the result of a lack of correlation between MSR and BAO abilities (and the much lower passing rate in the MSR compared to the BAO task) is not due to sample issues and proves repeatable, it would raise an interesting conceptual question about what we are measuring in these tasks and whether the BAO and MSR task both measure aspects of an underlying objective self‐awareness in nonhuman animals or not.

The concurrent emergence of both the ability to recognize oneself in a mirror and to perceive one's own body as the obstacle to action during human ontogenetic development suggests similar underlying developmental mechanisms, making BSA, as measured in the BAO tasks (Bullock and Lütkenhaus 1990; Moore et al. 2007), an indicator for the developing mental representation of self and conscious awareness of one's own body. In this case, the lack of correlation between the performances in the two tasks in great apes would indicate that MSR is a more conservative measure of an individual's self‐awareness abilities than the BAO task, which might result in a larger number of false negatives and might therefore not be the best indicator of self‐awareness on a population level. The BAO task might be more accessible for individuals, as it might be ecologically more relevant and more motivating, as, contrarily to the MSR task, the BAO task involved food rewards. The BAO task might consequently be more successful at detecting self‐awareness in nonhuman animals.

Conversely, as proposed by Moore and colleagues (2007) and Brownell and colleagues (2007), MSR and the ability to conceive one's own body as an obstacle might measure two distinct facets of objective self‐awareness, which might develop and manifest independently from each other in a species (Brownell et al. 2007; Moore et al. 2007). In this case, the performances in the MSR task and the BAO task could represent complementary measures when studying self‐awareness in nonhuman animals, allowing for the study of the different dimensions of self‐awareness. The understanding of own's own body weight as a hindrance to an action might constitute a more fundamental aspect of objective self‐awareness, which may explain the higher success rate in the BAO task.

Alternatively, the lack of correlation between the ability for MSR and the ability to perceive one's own body as an obstacle could also suggest that body awareness, as measured in this and other BAO tasks, might not necessarily require objective self‐awareness. Instead, these tasks could potentially be solved through more fundamental (potentially unconscious) mechanisms. This questions whether successfully completing such tasks may indicate an individual's possession of a body schema (i.e., a nonconscious experience of the body) rather than a body image (i.e., reflective knowledge about one's own body) (Gallagher 1986; Riva 2018 but see Pitron and De Vignemont 2017).

Although body schema and body image both relate to the perception and awareness of an individual's own body, they fundamentally differ in their underlying mechanisms and in their meaning for an individual's body representation. The body image relates to an individual's objective awareness, requires conscious processing and allows self‐reflection. The body schema, on the other hand, provides individuals with a sense of agency over their own body, but the processing can be conducted on an unconscious level as part of a subjective awareness. This ability is likely shared by all living beings (Morin 2011) as knowing the limits of one's own body, its relative size and weight appears to be abilities essential for survival in many species, and scaling errors (Brownell et al. 2007; DeLoache et al. 2004) could have detrimental fitness consequences (e.g., not choosing the right branches when moving through trees and risking a fall, or getting stuck in a hole i.e. too small to pass the entire body).

In nonhuman animals, evidence for some level of body awareness has been found in a variety of species ranging from hermit crabs that successfully find fitting shells (Krieger et al. 2020), and budgerigars that adapt their flight pattern to gap sizes (Schiffner et al. 2014), to snakes, rats, ferrets, crows, and dogs that successfully choose the right‐sized openings to pass through (Khvatov et al. 2019; Khvatov et al. 2021b, 2021a; Khvatov et al. 2021; Lenkei et al. 2020), and dogs and elephants that were both shown to understand their bodies as being obstacles to a task (Dale and Plotnik 2017; Lenkei et al. 2021). Four of these species— dogs, rats, hooded crows, and Asian elephants—have also been investigated for their responses to mirrors, (Plotnik et al. 2006; Smirnova et al. 2020; Yakura et al. 2018; Zazzo 1948, 1993), yet only the Asian elephant has also been show signs of mirror self‐recognition (Plotnik et al. 2006).

Disentangling the involvement of body image and body schema by implementing multiple complementary methodological approaches in the investigations of BSA in nonhuman animals coupled with a careful and parsimonious interpretation of the results of such studies, therefore appears to be a necessity for future investigations on this topic to gain a better understanding of how much BSA tasks inform us about an individual´s perceptual and conceptual knowledge of its own body.

The co‐emergence of the ability to conceive one's own body as an obstacle to an action and mirror self‐recognition in children would indicate that both of these abilities are the results of similar developmental processes related to the formation of a sense of self. Considering our results of a lack of correlation between the performances in the two tasks, as well as past findings in humans and nonhuman animals, the BAO tasks might represent an interesting new avenue for the study of other dimensions of self‐awareness once simpler task‐solving mechanisms can be excluded. Alternatively, these results might indicate that the mechanisms underlying mirror self‐recognition and the understanding of one's own body as an obstacle might be independent and require further clarification on whether the processes involved in solving the BAO task necessarily require a form of objective self‐awareness.

## Author Contributions

**Lisa‐Claire Vanhooland:** conceptualization (equal), data curation (lead), formal analysis (lead), funding acquisition (lead), investigation (lead), methodology (lead), project administration (lead), supervision (supporting), visualization (equal), writing – original draft (lead). **Constanze Mager:** resources (equal). **Aurora Teuben:** data curation (equal), investigation (equal). **Thomas Bugnyar:** resources (supporting), supervision (equal), writing – review and editing (supporting). **Jorg J M Massen:** conceptualization (equal); methodology (equal), resources (equal), supervision (lead), writing – original draft (supporting), writing – review and editing (supporting).

## Conflicts of Interest

The authors declare no conflicts of interest.

## Supporting information

Supporting information.

Supporting information.

Supporting information.

Supporting information.

## Data Availability

The data of this study are openly available at DOI: 10.6084/m9. figshare.24517462.
