# Supplementary material for: Comparing the Performances of Chimpanzees (Pan troglodytes) and Gorillas (Gorilla gorilla gorilla) in Two Self‐Awareness Tasks
Source: Am J Primatol. 2025 Mar 6;87(3):e70010. doi: 10.1002/ajp.70010 (PMC11886032; doi:10.1002/ajp.70010)
Supplement: Supplementary file 1 — Supporting information. [file AJP-87-e70010-s002.docx]

**Supplementary materials**

**Table S1.** Subject specifications

| Species | Name | Sex | YoB | Origin |
| --- | --- | --- | --- | --- |
| Chimpanzee | Erika | Female | 1992 | Givskud Zoo |
| Chimpanzee | Fons | Male | 1975 | Royal Burgers´ Zoo |
| Chimpanzee | Gaby | Female | 1984 | Royal Burgers´ Zoo |
| Chimpanzee | Geisha | Female | 1993 | Royal Burgers´ Zoo |
| Chimpanzee | Ghineau | Male | 2005 | Royal Burgers´ Zoo |
| Chimpanzee | Giambo | Male | 1989 | Royal Burgers´ Zoo |
| Chimpanzee | Jimmie | Female | 1960 | Wild caught |
| Chimpanzee | Jing | Male | 1981 | Royal Burgers´ Zoo |
| Chimpanzee | Moni | Female | 1989 | Kittemberger Kalman |
| Chimpanzee | Moniek | Female | 1977 | Royal Burgers´ Zoo |
| Chimpanzee | Morami | Female | 1987 | Royal Burgers´ Zoo |
| Chimpanzee | Raimee | Female | 1999 | Royal Burgers´ Zoo |
| Chimpanzee | Roosje | Female | 1979 | Royal Burgers´ Zoo |
| Chimpanzee | Tesua | Female | 1986 | Royal Burgers´ Zoo |
| Chimpanzee | Tushi | Female | 1992 | Royal Burgers´ Zoo |
| Gorilla | Bauwi | Male | 1989 | Apenheul Primate Park |
| Gorilla | N´Gayla | Female | 1993 | Royal Burgers´ Zoo |
| Gorilla | N´irale | Female | 2013 | Royal Burgers´ Zoo |
| Gorilla | N´Aika | Female | 2005 | Royal Burgers´ Zoo |
| Gorilla | N´kato | Male | 2013 | Royal Burgers´ Zoo |
| Gorilla | N´hasa | Female | 2013 | Royal Burgers´ Zoo |
| Gorilla | Nimba | Female | 1999 | Barcelona Zoo |
| Gorilla | Nakouh | Male | 2009 | Royal Burgers´ Zoo |
| Gorilla | Nukta | Male | 2013 | Royal Burgers´ Zoo |
| Gorilla | Makua | Female | 2004 | Zoologischer Garten Berlin |
| Gorilla | Madiba | Male | 2013 | Royal Burgers´ Zoo |

**Table S2**. Ethogram of mirror-related behaviors (behavior type coded as (E) for behaviors coded as events and (D) for behaviors coded as durations)

| Behavioral category | Type | Name of behaviors | Description of the behaviors |
| --- | --- | --- | --- |
| **Social behaviors**  **directed at the object** | (E) | Affiliative | E.g. Lip smacking: Rapid closing and opening of the mouth and lips while looking in the mirror |
|  | (E) | Agonistic behaviors | Attack, charging, Aggressive slap of the object, bristling (i.e. hair erection while looking at the object), grimacing |
|  | (E) | Vocalization | Creating sounds by use of the vocal cords. Can be affiliative or aggressive. |
| **Physical contact** | (D) | Hold | Individual holds the object in its hands or mouth |
|  | (D) | Transport | Individual is carrying the object in its mouth, hand or on its the back while moving |
|  | (D) | Stepping on object | Individual steps on the object with its hand or foot while walking over it or stands on it. |
| **Explorative behaviors** | (E) | Sniffing | Individual holds the object against its nostrils |
|  | (E) | Throwing | Individual throws the objects in the air |
|  | (E) | Licking | Individual licks the surface of the object |
|  | (E) | Use as tool | Individual uses the object as a tool e.g., for digging |
|  | (E) | Knocking | Individual knocks against the object with its knuckles |
|  | (E) | Scratching | Individual scratches the surface of the object |
|  | (E) | Biting | Individual puts the object in its mouth and bites down on it |
|  | (E) | Tapping | Individual performs light taps against the object with its fingers |
|  | (E) | Slapping | Individual slaps object with the palm of its hand |
| **Close inspection** | (D) |  | Individual is holding the object close to its eyes and looking into it / through it (control) or object in on the ground and the animals looks closely at it |
| **Contingency checking behaviour*** | (E) | Rocking^*^ | Contingent locomotion: repetitive movement from left to right or from front to back |
|  | (E) | Peekaboo^*^ | Individual moves in and out of sight of the mirror within 5 seconds |
|  | (E) | Sticking out tongue^*^ | Individual sticks out tongue while looking in the mirror |
| **Self-directed behaviors** | (E) | Autogrooming | Individual is grooming itself while touching the object |
|  | (E) | Touching face | Individual touches any part of its face while touching the object |
|  | (E) | Touching ano-genital region^*^ | Individual touches its genital or anal region while touching the object |
|  | (E) | Scratching | Individual scratches a part of its body while touching the object |
|  | (E) | Open mouth^*^ | Individual keeps its mouth wide open while touching at the object |
| **Mirror-guided self-directed behaviors** | (E) | Mirror-directed self-grooming | Individual is grooming itself while looking in the object |
|  | (E) | Touching face | Individual touches any part of its face while looking at the object |
|  | (E) | Touching ano-genital region^*^ | Individual touches its genital or anal region while looking at the object |
|  | (E) | Scratching | Individual scratches a part of its body while looking at the object |
|  | (E) | Open mouth | Individual keeps its mouth wide open while looking at the object (could allow exploration of the inside its mouth in the mirror condition) |
| **Other** | (D) | Use as tool | Individual uses object as a tool e.g., for digging |
|  | (E) | Reaching | Individual extends hand towards the object |
|  | (E) | Pick up | Individual picks up the object from the ground |
|  | (E) | Drop mirror | Individual drops or puts down the object the object on the ground |
|  | (D) | Sitting in close proximity | Individual is in reaching distance of the object |
|  | (D) | Touching | Individual is touching the object with a part of its body without holding or interacting with the object |
|  | (E) | Showing teeth | Individual retracts lips to expose teeth while looking in the mirror |
|  | (E) | Yawn | Involves mouth opening, deep inspiration, brief apnea, and slow expiration while looking at the object the mirror |

^*^Behavior or behavioral category that was never exhibited during the experiment

**Table S3**. Ethogram used for the Body self-awareness experiment (behavior type coded as (E) for behaviors coded as events and (D) for behaviors coded as durations)

| Category | Type | Behavior | Description |
| --- | --- | --- | --- |
| Box proximity | (D) | Next to the box | Individual sit in very close proximity (grabbing distance) of box |
|  | (D) | On top of the box (lid open) | Individual is sitting on the box while the lid is open |
|  | (D) | On top of the box (lid closed) | Individual is sitting on top of the box while the lid is closed |
| Box Interactions | (E) | Open lid (success) | Individual successfully opens the lid |
|  | (E) | Open lid (failed attempt) | Individual pulls on the lid but does not manage to open it |
|  | (E) | Reach into Box for food | Individual reaches into the box to grab food |

***
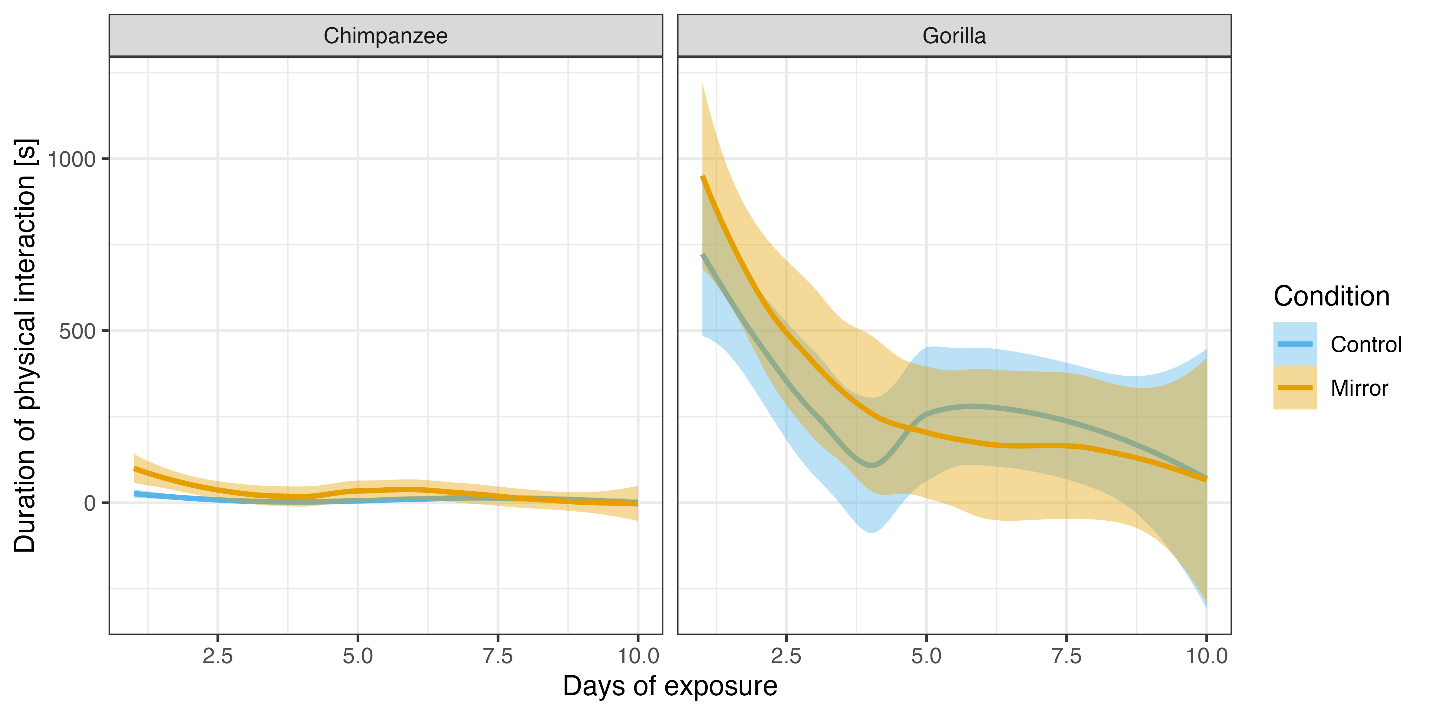
***

**Figure S1.** Summed duration of the time individuals engaged in physical contact (i.e., holding or transporting) with the objects (mirror and control) over the 10 days of exposure (days 1-5 correspond to the first week, and days 6-10 to the second week of exposure) of each condition for the chimpanzees and the gorillas

**Table S4.** Descriptive statistics on the number of unsuccessful attempts to open the test box and the number of individuals with at least one failed attempt for all three conditions, the lowest and highest number of unsuccessful attempts by individuals (Min-Max) and the average (mean ± SD) efficiency of individuals in their first attempt to open the box across all trials (Total Efficiency).

| Condition | Species | Sum Failed attempts (Number of Individuals with at least one error) | Min-Max | Total Efficiency  Mean (SD) |
| --- | --- | --- | --- | --- |
| Ground | Chimpanzees | 2 (2) | 0-1 | 0.998 (0.025) |
|  | Gorillas | 2 (2) | 0-1 | 0.999 (0.025) |
| Suspended on ropes | Chimpanzees | 4 (3) | 0-2 | 0.997 (0.092) |
|  | Gorillas | 0 (0) | 0 | 1 (0) |
| Pole | Chimpanzees | 19 (6) | 0-7 | 0.983 (0.037) |
|  | Gorillas | 25 (10) | 0-9 | 0.967 (0.124) |

**Table S5.** Number of social behaviors, self-directed behaviors exhibited in close proximity to an object, and mirror-guided self-directed behaviors (i.e., self-directed behaviors exhibited while looking directly at the object) performed by each species in each condition (the number in parentheses indicates the number of individuals observed performing these behaviors)

| Behavior | Mirror condition | | Control condition | |
| --- | --- | --- | --- | --- |
|  | Chimpanzees | Gorillas | Chimpanzees | Gorillas |
| Social behaviors | 7 (2) | 0 | 2 (1) | 1 (1) |
| Self-directed behaviors in proximity of the object | 41 (7) | 23 (5) | 1 (1) | 16 (4) |
| Mirror-guided self-directed behaviors | 26 (2) | 1 (1) | 0 | 0 |
